# Supplementary material for: Research on rapier loom fault system based on cloud-side collaboration
Source: PLoS One. 2021 Dec 31;16(12):e0260888. doi: 10.1371/journal.pone.0260888 (PMC8719711; doi:10.1371/journal.pone.0260888)
Supplement: S1 File — (DOCX) [file pone.0260888.s001.docx]

In this paper, the a priori probability of loom fault results and the causal relationship strength of fault cause set are obtained through the analysis of nearly 500 loom fault information collected on site, as shown in Figure 1. Number


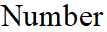

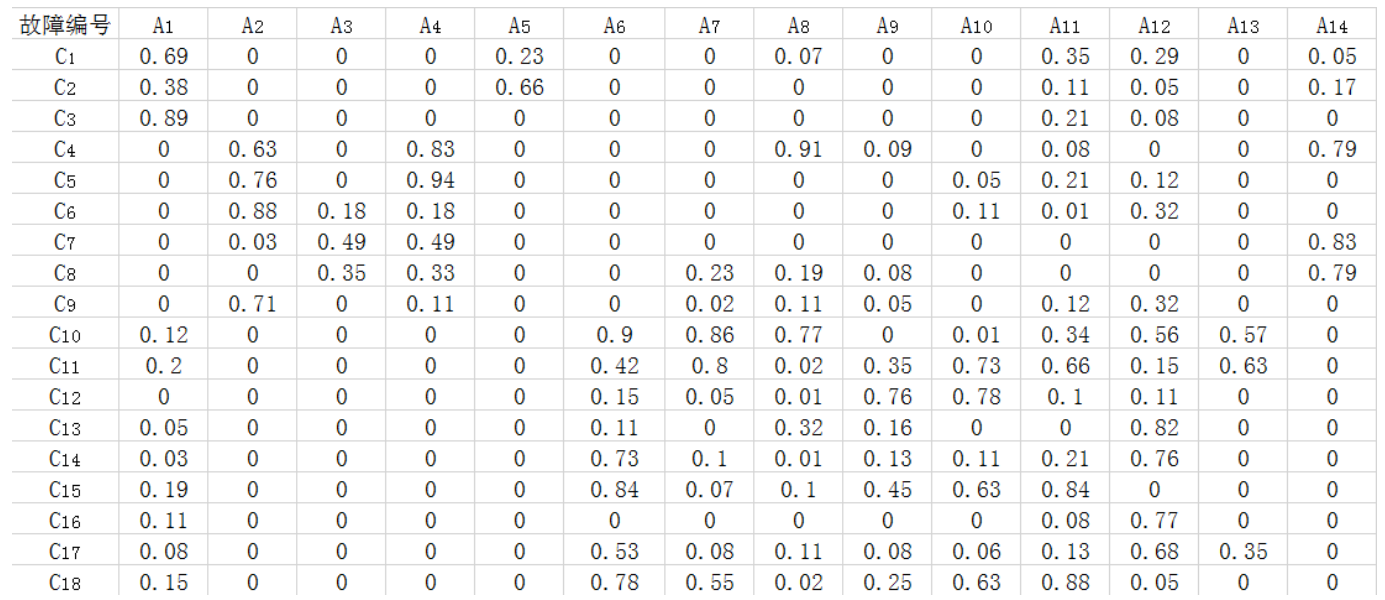


Figure1 Loom failure causality strength table

The table in the figure above is equivalent to the decision table when using rough set. Discretize the values in the table. Then the loom fault diagnosis decision table is shown in table1.

Table 1 Loom Failure Decision Table

| Number | A_1_ | A_2_ | A_3_ | A_4_ | A_5_ | A_6_ | A_7_ | A_8_ | A_9_ | A_10_ | A_11_ | A_12_ | A_13_ | A_14_ |
| --- | --- | --- | --- | --- | --- | --- | --- | --- | --- | --- | --- | --- | --- | --- |
| C_1_ | 3 | 0 | 0 | 0 | 0 | 0 | 0 | 1 | 0 | 0 | 1 | 2 | 0 | 1 |
| C_2_ | 2 | 0 | 0 | 0 | 2 | 0 | 0 | 0 | 0 | 0 | 1 | 1 | 0 | 1 |
| C_3_ | 4 | 0 | 0 | 0 | 4 | 0 | 0 | 0 | 0 | 0 | 1 | 1 | 0 | 0 |
| C_4_ | 0 | 2 | 0 | 1 | 0 | 0 | 0 | 1 | 1 | 0 | 1 | 0 | 0 | 4 |
| C_5_ | 0 | 4 | 0 | 4 | 0 | 0 | 0 | 0 | 0 | 1 | 1 | 2 | 0 | 0 |
| C_6_ | 0 | 4 | 4 | 1 | 0 | 0 | 0 | 0 | 0 | 1 | 1 | 2 | 0 | 0 |
| C_7_ | 0 | 1 | 1 | 1 | 0 | 0 | 0 | 0 | 0 | 0 | 0 | 0 | 0 | 4 |
| C_8_ | 0 | 0 | 2 | 2 | 0 | 0 | 0 | 0 | 0 | 0 | 0 | 0 | 0 | 3 |
| C_9_ | 0 | 3 | 0 | 1 | 0 | 0 | 0 | 1 | 1 | 0 | 1 | 2 | 0 | 0 |
| C_10_ | 1 | 0 | 0 | 0 | 0 | 1 | 4 | 4 | 0 | 1 | 2 | 3 | 3 | 0 |
| C_11_ | 1 | 0 | 0 | 0 | 0 | 2 | 4 | 1 | 2 | 3 | 3 | 2 | 3 | 0 |
| C_12_ | 0 | 0 | 0 | 0 | 0 | 1 | 1 | 1 | 4 | 4 | 1 | 1 | 0 | 0 |
| C_13_ | 1 | 0 | 0 | 0 | 0 | 0 | 0 | 0 | 0 | 0 | 1 | 4 | 0 | 0 |
| C_14_ | 1 | 0 | 0 | 0 | 0 | 3 | 1 | 1 | 1 | 1 | 1 | 4 | 2 | 0 |
| C_15_ | 1 | 0 | 0 | 0 | 0 | 4 | 3 | 1 | 2 | 3 | 4 | 1 | 0 | 0 |
| C_16_ | 0 | 0 | 0 | 0 | 0 | 4 | 1 | 1 | 1 | 3 | 4 | 1 | 4 | 0 |
| C_17_ | 0 | 0 | 1 | 1 | 0 | 0 | 0 | 0 | 0 | 2 | 1 | 3 | 0 | 0 |
| C_18_ | 0 | 0 | 0 | 0 | 0 | 0 | 1 | 1 | 1 | 1 | 1 | 2 | 0 | 0 |

Applying rough set theory to attribute reduction in table 3.10, two minimum attribute sets are obtained:{A_1_,A_2_,A_3_,A_6_,A_8_,A_9_,A_11_,A_12_,A_13_,A_14_},and{A_1_,A_2_,A_3_,A_5_,A_6_,A_8_,A_9_,A_12_,A_13_,A_14_}.A_11_ fault is not easy to obtain, so B is selected as the minimum fault. The minimum reduction is shown in table 2.

Table 2 Minimum Reduction

| Number | A_1_ | A_2_ | A_3_ | A_5_ | A_6_ | A_8_ | A_9_ | A_12_ | A_13_ | A_14_ |
| --- | --- | --- | --- | --- | --- | --- | --- | --- | --- | --- |
| C_1_ | 3 | 0 | 0 | 0 | 0 | 1 | 0 | 2 | 0 | 1 |
| C_2_ | 2 | 0 | 0 | 2 | 0 | 0 | 0 | 1 | 0 | 1 |
| C_3_ | 4 | 0 | 0 | 4 | 0 | 0 | 0 | 1 | 0 | 0 |
| C_4_ | 0 | 2 | 0 | 0 | 0 | 1 | 1 | 0 | 0 | 4 |
| C_5_ | 0 | 4 | 0 | 0 | 0 | 0 | 0 | 2 | 0 | 0 |
| C_6_ | 0 | 4 | 4 | 0 | 0 | 0 | 0 | 2 | 0 | 0 |
| C_7_ | 0 | 1 | 1 | 0 | 0 | 0 | 0 | 0 | 0 | 4 |
| C_8_ | 0 | 0 | 2 | 0 | 0 | 0 | 0 | 0 | 0 | 3 |
| C_9_ | 0 | 3 | 0 | 0 | 0 | 1 | 1 | 2 | 0 | 0 |
| C_10_ | 1 | 0 | 0 | 0 | 1 | 4 | 0 | 3 | 3 | 0 |
| C_11_ | 1 | 0 | 0 | 0 | 2 | 1 | 2 | 2 | 3 | 0 |
| C_12_ | 0 | 0 | 0 | 0 | 1 | 1 | 4 | 1 | 0 | 0 |
| C_13_ | 1 | 0 | 0 | 0 | 0 | 0 | 0 | 4 | 0 | 0 |
| C_14_ | 1 | 0 | 0 | 0 | 3 | 1 | 1 | 4 | 2 | 0 |
| C_15_ | 1 | 0 | 0 | 0 | 4 | 1 | 2 | 1 | 0 | 0 |
| C_16_ | 0 | 0 | 0 | 0 | 4 | 1 | 1 | 1 | 4 | 0 |
| C_17_ | 0 | 0 | 1 | 0 | 0 | 0 | 0 | 3 | 0 | 0 |
| C_18_ | 0 | 0 | 0 | 0 | 0 | 1 | 1 | 2 | 0 | 0 |

According to the relationship between fault causes and fault results in table 2, a network model based on Nb (naive Bayes) is constructed, as shown in Figure 2.


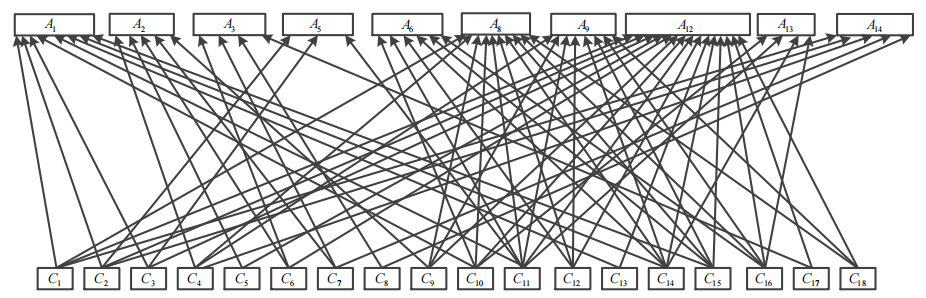


Figure 2 Lobe fault Bayesian model

Based on the 500 loom fault data collected on site, 300 of them are used as the learning and training samples of wake simulation, and the other 200 fault data are used as the fault diagnosis and verification samples. During actual loom data acquisition, due to the lack of data due to the complex field environment, the sample training is carried out under the condition of incomplete data. Among them, the collected loom fault data are classified according to the fault type, and the occurrence times of this type are summarized, as shown in Table 3.

Table 3 Loom Failure Data Sheet

| Serial number | Number | A_1_ | A_2_ | A_3_ | A_4_ | A_5_ | A_6_ | A_7_ | A_8_ | A_9_ | A_10_ | A_11_ | A_12_ | A_13_ | A_14_ | Quantity |
| --- | --- | --- | --- | --- | --- | --- | --- | --- | --- | --- | --- | --- | --- | --- | --- | --- |
| 1 | C_1_ | 3 | 0 | 0 | 0 | * | 0 | 0 | 1 | 0 | 0 | 1 | * | 0 | 1 | 133 |
| 2 | C_2_ | 2 | 0 | 0 | 0 | 3 | 0 | 0 | 0 | 0 | 0 | 0 | 1 | 0 | 1 | 2 |
| 3 | C_3_ | 4 | 1 | 1 | 0 | * | 0 | 0 | 1 | 0 | 0 | * | 0 | 0 | 0 | 16 |
| 4 | C_4_ | 0 | 2 | 1 | 0 | 0 | 0 | 1 | 0 | 0 | 0 | 1 | * | 0 | 4 | 13 |
| 5 | C_5_ | 0 | 4 | 0 | 4 | 0 | 0 | 0 | 0 | 0 | * | 1 | 2 | 0 | 0 | 66 |
| 6 | C_6_ | 0 | 4 | 4 | * | 0 | 0 | 0 | 0 | 0 | 1 | 1 | 2 | 0 | 0 | 59 |
| 7 | C_7_ | 0 | * | 1 | * | 0 | 0 | 0 | 0 | 1 | 0 | 0 | 1 | 0 | 4 | 3 |
| 8 | C_8_ | 0 | 0 | 2 | 2 | 0 | 0 | 0 | 0 | 0 | 0 | 0 | 0 | 0 | 3 | 6 |
| 9 | C_9_ | 0 | 3 | 0 | 1 | 0 | 0 | 0 | 1 | * | 0 | * | 2 | 0 | 0 | 140 |
| 10 | C_10_ | * | 0 | 0 | 0 | 1 | 0 | 4 | 4 | 0 | 1 | 2 | 0 | 3 | 0 | 6 |
| 11 | C_11_ | 1 | 0 | 0 | 0 | 0 | 2 | 4 | 1 | * | 0 | 3 | 2 | 3 | 0 | 4 |
| 12 | C_12_ | 0 | 0 | 0 | 0 | 0 | 0 | 0 | * | 4 | 4 | 0 | 0 | 1 | 0 | 2 |
| 13 | C_13_ | 0 | 0 | * | 1 | 0 | 1 | 0 | 2 | 1 | 0 | 1 | 4 | 0 | 0 | 39 |
| 14 | C_14_ | 0 | 0 | 0 | 0 | 0 | 3 | 1 | 0 | 0 | 1 | 0 | * | 2 | 0 | 1 |
| 15 | C_15_ | 0 | 0 | 0 | 0 | 0 | * | 3 | 0 | 0 | 3 | 4 | 0 | 0 | 0 | 4 |
| 16 | C_16_ | 0 | * | 0 | 0 | 0 | 4 | 1 | * | 1 | 3 | 4 | 1 | 4 | 0 | 3 |
| 17 | C_17_ | 0 | 0 | 1 | 1 | 0 | 0 | 0 | 0 | 0 | 2 | * | 3 | 0 | 0 | 2 |
| 18 | C_18_ | 0 | 0 | * | 0 | 0 | 0 | 1 | 0 | 1 | 0 | 1 | 2 | 0 | 0 | 1 |

Where "*" in table 3 represents the missing loom attributes.
